# Current role of prostate-specific membrane antigen-based imaging and radioligand therapy in castration-resistant prostate cancer

**DOI:** 10.3389/fcell.2022.958180

**Published:** 2022-08-12

**Authors:** Jiaxian Chen, Lin Qi, Yongxiang Tang, Guyu Tang, Yu Gan, Yi Cai

**Affiliations:** ^1^ Department of Urology, National Clinical Research Center for Geriatric Disorders, Xiangya Hospital, Central South University, Changsha, Hunan Province, China; ^2^ Department of PET Center, National Clinical Research Center for Geriatric Disorders, Xiangya Hospital, Central South University, Changsha, Hunan Province, China

**Keywords:** prostate-specific membrane antigen, castration-resistant prostate cancer, positron emission tomography, radioligand therapy, theranostics

## Abstract

Castration-resistant prostate cancer (CRPC) is a therapy-resistant and lethal form of prostate cancer as well as a therapeutic challenge. Prostate-specific membrane antigen (PSMA) has been proved as a promising molecular target for optimizing the theranostics for CRPC patients. When combined with PSMA radiotracers, novel molecular imaging techniques such as positron emission tomography (PET) can provide more accurate and expedient identification of metastases when compared with conventional imaging techniques. Based on the PSMA-based PET scans, the accurate visualization of local and disseminative lesions may help in metastasis-directed therapy. Moreover, the combination of ^68^Ga-labeled PSMA-based PET imaging and radiotherapy using PSMA radioligand therapy (RLT) becomes a novel treatment option for CRPC patients. The existing studies have demonstrated this therapeutic strategy as an effective and well-tolerated therapy among CRPC patients. PSMA-based PET imaging can accurately detect CRPC lesions and describe their molecular features with quantitative parameters, which can be used to select the best choice of treatments, monitor the response, and predict the outcome of RLT. This review discussed the current and potential role of PSMA‐based imaging and RLT in the diagnosis, treatment, and prediction of prognosis of CRPC.

## Introduction

Prostate cancer (PCa) has 1.3 million new cases each year worldwide, making it the second most prevalent cancer among men (Sung et al., 2021). Since Huggins and Hodges discovered the hormone dependence of PCa in 1941, androgen deprivation therapy (ADT) has become the mainstay for treating PCa (Hansson et al., 2016). Most of the PCa tumors are initially sensitive to ADT but develop resistance ultimately and transform into castration-resistant prostate cancer (CRPC), which represents the lethal form of PCa (Shafi et al., 2013; He et al., 2020).

The diagnosis of CRPC depends on the occurrence of biochemical and/or radiological progression in a castration environment (castrate serum testosterone < 50 ng/dl or 1.7 nmol/l). At present, conventional imaging modalities (CIMs), such as magnetic resonance imaging (MRI), abdominopelvic computed tomography (CT), and whole-body bone scans (BS) are still recommended as the standard imaging techniques to evaluate radiological progression. However, CIMs may delay the diagnosis of CRPC due to the lack of accuracy in identifying metastases, therefore postponing the necessary therapy switch. More powerful imaging tools are needed to identify CRPC earlier and timely to trigger subsequent treatments.

Over the last decades, chemotherapeutic drugs, such as cabazitaxel and docetaxel, second-generation inhibitors of the androgen-receptor signaling, such as enzalutamide and abiraterone acetate, as well as radium-223 have improved the clinical benefits of patients with CRPC. However, as the disease progresses, these treatments eventually become useless. Furthermore, the lack of reliable markers to well predict the outcome of CRPC patients remains a challenge in the management of CRPC patients.

In the last two decades, PSMA (a 750–amino acid type II transmembrane glycoprotein) has been demonstrated as a potential diagnostic and therapeutic target for PCa (O'Keefe et al., 2018). It has several advantages when compared with the other molecular markers used in PCa. First, PSMA is highly expressed in PCa, bone metastasis, lymph node, and nearly all stages of the disease but has a low expression in normal prostatic tissues (Sweat et al., 1998). As reported, the PSMA expression in PCa can be elevated by 100–1,000 folds when compared with the benign prostatic tissues (Silver et al., 1997). Second, an increase in the pathologic stage, tumor grade, and biochemical recurrence can enhance PSMA expression. In particular, PSMA expression increases extremely when PCa tumors progress into a castration‐resistant stage (Paschalis et al., 2019). Third, the internalization of PSMA by tumor cells may help in targeted therapies (Nguyen et al., 2016).

Of late, in clinical practice, PET scans are usually combined with cross-sectional imagings like CT to form the positron emission CT (PET/CT). Therefore, PET/CT that combines the molecular advantages of PET and morphologic features of CT is superior to the CIMs for the diagnosis and monitoring of various cancer types including PCa. Moreover, when combined with PSMA radiotracers, PET/CT showed remarkable sensitivity and specificity in the field of PCa (Fendler et al., 2019a). Of late, the US Food and Drug Administration has approved ^68^Ga-PSMA-11 (Carlucci et al., 2021) and ^18^F-DCFPyL (Song et al., 2022) as novel PET imaging tracers for PCa. Given the superiority compared with CIMs, PMSA-based PET/CT is expected to optimize the diagnosis of CRPC.

**FIGURE 1:**
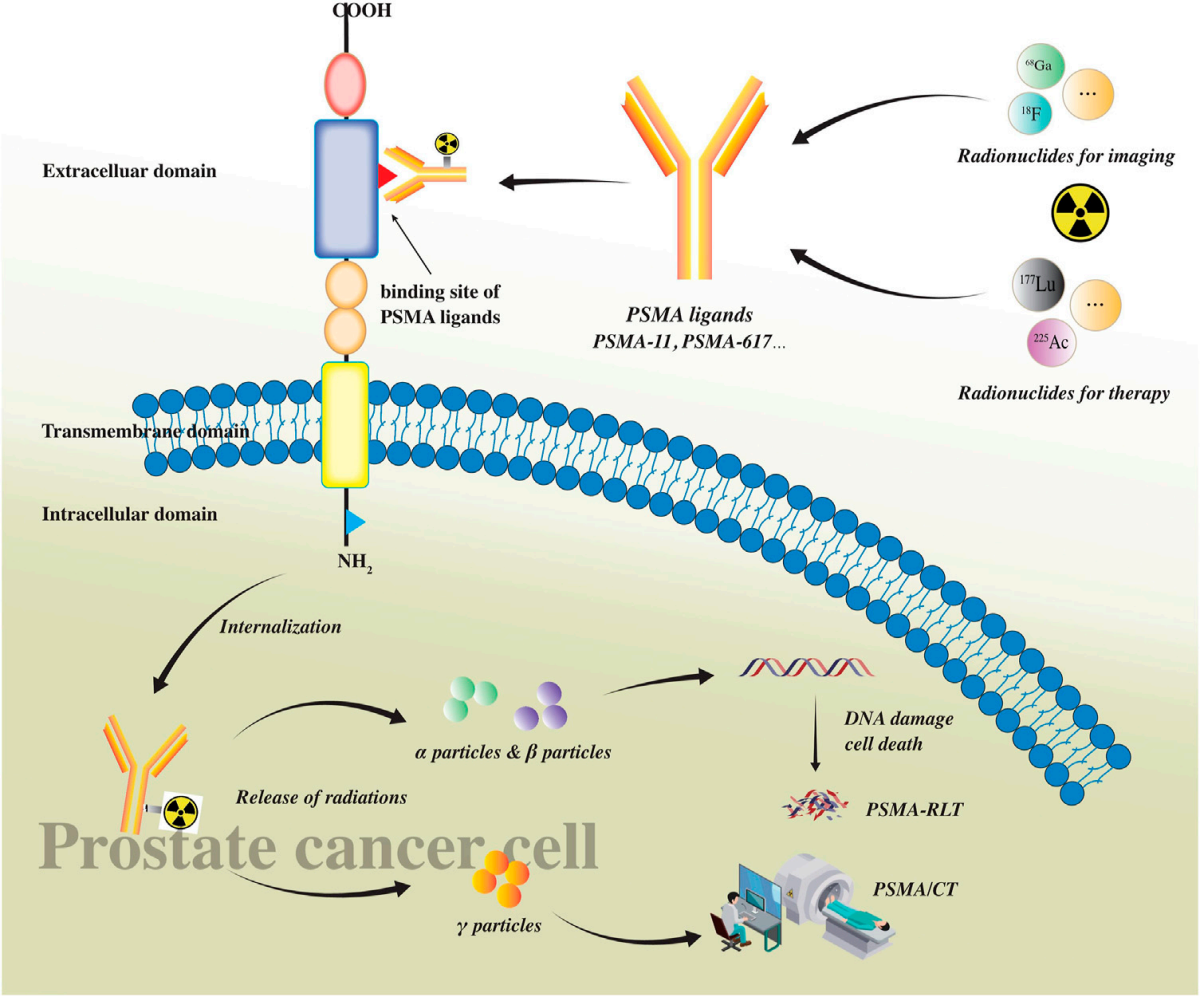
PSMA as a transmembrane glycoprotein consists of three parts: intracellular, transmembrane, and extracellular domain. There is a ligand binding site in the extracellular domain where ligands bind with PSMA on prostate cancer cells. PSMA ligands (such as PSMA-11 and PSMA-617) can be labeled with radionuclides for imaging (such as ^68^Ga and ^18^F) and/or therapy (such as ^177^Lu and ^225^Ac). After radiolabeled PSMA ligands bind with the extracellular ligand binding site of PSMA, they would be internalized into prostate cancer cells, releasing different particles. Alpha(α) and beta(β) particles can cause DNA damage leading to cancer cell death and be used in PSMA RLT. Gamma (*γ*) particles can be detected using PET scans and be used in PET/CT imaging.

Radioligand therapy (RLT), targeting the PSMA, showed desired antitumor effects by combining the therapeutic radionuclides with the PSMA ligands and delivering a high-radiation dose to the targeted tumor cells (Sgouros et al., 2020). Alpha- or beta-emitting radionuclides, such as Actinium-225 (^225^Ac) and Lutetium-177 (^177^Lu), respectively, can be used to label PSMA ligands. Figure 1 shows how radiolabeled ligands bind with PSMA on PCa cells and serve as imaging and radioligands therapeutic tools. Moreover, quantitative parameters derived from PSMA-based PET/CT alone or from the combination with 18-fluorodeoxyglucose (^18^F-FDG) PET/CT have been proven to be predictive of the outcome of patients before receiving RLT, thereby improving the selection of the best choice of management option and drug at an individual level.

CRPC has always been a bottleneck for PCa, which calls for continuous exploration. Based on the current research, the emergence of PSMA-based PET scans and RLT has brought hope to tackle the problems in the diagnostic, therapeutic, and prognostic field of CRPC. This review aims to discuss the current role of PSMA‐based imaging and RLT in CRPC.

### Diagnostic role of prostate-specific membrane antigen-based imaging in castration-resistant prostate cancer patients

The biochemical progression and/or radiological progression of PCa with castrated serum testosterone < 50 ng/dl or 1.7 nmol/l refer to as CRPC. CRPC can be divided into metastatic CRPC (mCRPC) and nonmetastatic CRPC (nmCRPC) according to the distant metastasis state based on the reports of CIMs. The biochemical progression is defined as three consecutive increases in the prostate-specific antigen (PSA), which results in a two-time 50% increase from the baseline PSA level, reaching > 2 ng/ml (Cornford et al., 2021) or PSA > 1 ng/ml (Scher et al., 2016). On the other hand, conventional cross-sectional imagings by either CT or MRI and BS are still recommended as standard staging tools to evaluate radiological progression in patients suspected of CRPC (Cornford et al., 2021).

### Superiority of prostate-specific membrane antigen-based imaging over conventional imaging modalities

**FIGURE 2:**
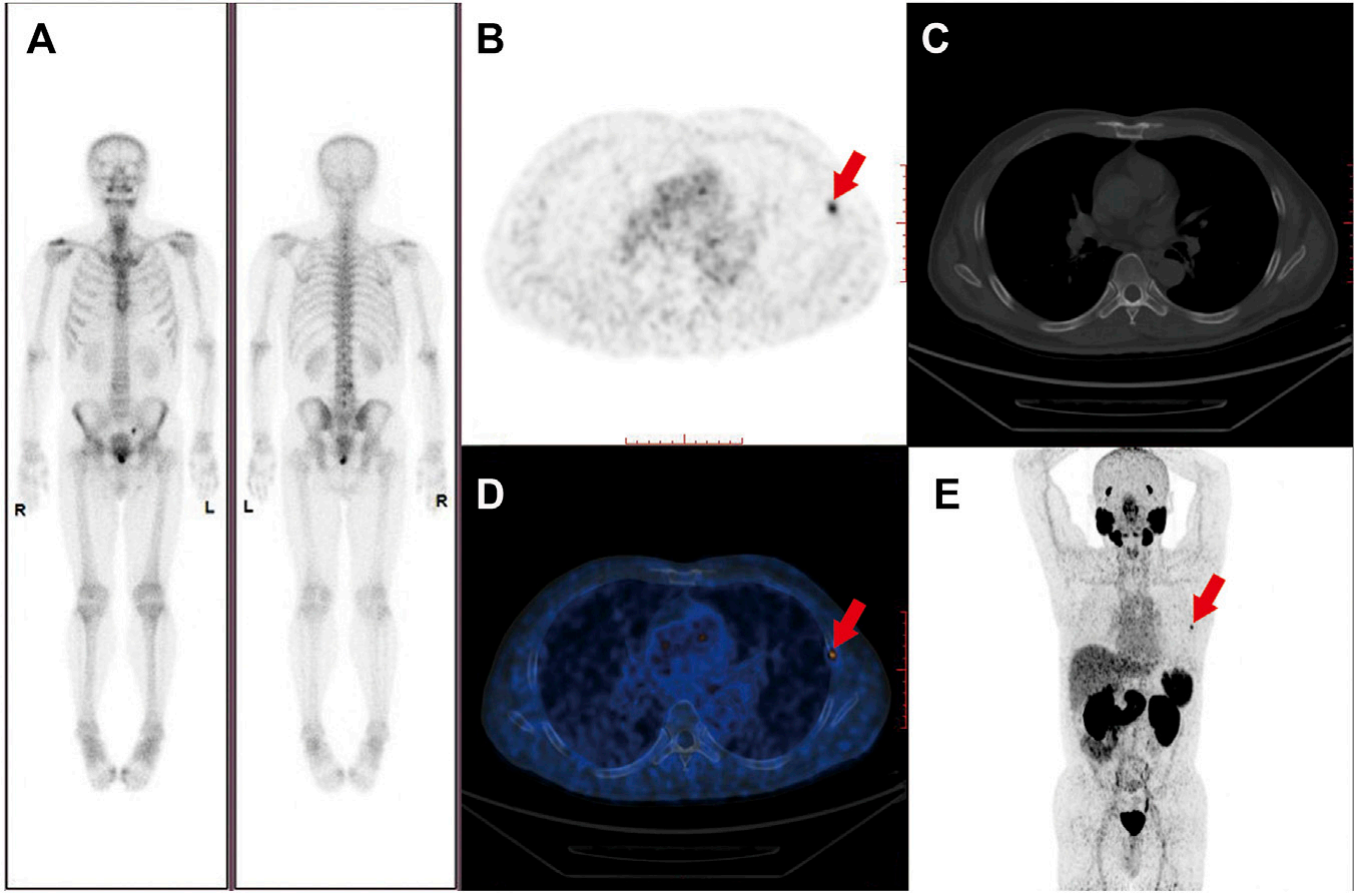
Imaging of a 56-year-old patient with Gleason score 7 prostate cancer and rising PSA following laparoscopic prostatectomy. PSA was 1.51 ng/ml at the time of the PET scan. PSMA-PET maximum-intensity projection, axial slides, and axial fused PET/CT revealed metastasis bone lesion on the left fifth rib (where the red arrows pointed to) with a SUVmax of 5.1 **(B,D,E)**. The lesion was not detected on the **(A)** Bone scan and **(C)** CT scan performed simultaneously.

The conventional imaging techniques, such as CT and MRI, have a limited accuracy to detect metastatic PCa lesions in lymph nodes and bones. In a pooled analysis of 18 clinical studies, CT showed a limited sensitivity [42% (95% CI 26–56%)] for the detection of metastatic lymph nodes (Hovels et al., 2008). Due to the low specificity and sensitivity, conventional imaging techniques seem unable to provide an accurate overall tumor burden. In a different way, the PSMA-PET/CT has demonstrated an unprecedented accuracy in the initial staging or restaging when the biochemical relapse of PCa occurs after treatments with curative intent. A 27% greater accuracy of the PSMA-PET/CT was found in the Phase 3 clinical trial by Hofman et al. when compared with the conventional imaging techniques, which translated into modulations in the management (Hofman et al., 2020). At present, there is also solid evidence of the restaging efficacy of PSMA-PET/CT in men with CRPC (Pandit-Taskar et al., 2015; Pyka et al., 2016; Rowe et al., 2016). Figure 2 showed the imagings of a 56-year-old patient with an initial diagnosis as nmCRPC according to the results from CIMs, for whom PSMA-PET detected evidence of distant metastatic lesions on the left fifth rib.

In 2019, Frendler et al. employed the PSMA-based PET to evaluate tumor burden in 200 patients who were diagnosed as nmCRPC via CIMs. However, based on the reevaluation of PSMA-based PET scans, 55% showed evidence of distant metastases (M1 stage) leading to a stage migration from nmCRPC to mCRPC (Fendler et al., 2019b). Likewise, Weber et al. confirmed that the PSMA-based PET/CT had a higher detection rate when compared with that of CIMs among the 55 patients with early CRPC (serum PSA ≤ 3 ng/ml) (Weber et al., 2021). Fourquet et al. analyzed a total of 30 patients with an increase in their serum PSA levels after medical castration; as a result, the PSMA-based PET scans got positive findings in all the patients with a serum PSA level > 2 ng/ml (20/30) and in seven patients (7/30) with a PSA level < 2 ng/ml. In the end, the reevaluation of PET/CT affected the disease management in approximately 70% of the patients (Fourquet et al., 2020).

### Potential clinical impacts on the management of castration-resistant prostate cancer after accurate diagnosis via prostate-specific membrane antigen-based imaging

At present, the shift from hormone-sensitive prostate cancer (HSPC) to CRPC is diagnosed based on the PSA progression and radiological progression evaluated by CIMs according to the Response Evaluation Criteria in Solid Tumors (RECIST 1.1) (Schwartz et al., 2016). In consideration of the limitations of PSA, such as unspecific fluctuations in the low detection range, and given the poor diagnostic capability of CIMs especially when PSA is examined at a low level, the current definition of CRPC seems outdated. It can be inferred that for the HSPC patients who are receiving the ADT, the addition of PSMA-PET/CT follow-up can reveal a castration resistance state earlier than PSA and CIMs and trigger a treatment change ahead of time, which provides a possibility to slow down the deterioration of the disease and improve the prognosis. Nevertheless, a new response evaluation criteria to assess radiological progression via PSMA-PET/CT calls for further research (Fanti et al., 2020).

The higher sensitivity of PSMA-based PET when compared with the CIMs caused the earlier detection of metastases among the PCa patients, leading to a stage transition from nmCRPC to mCRPC in almost half of the patients (Fendler et al., 2019b; Weber et al., 2021). After the SPARTAN trial results were published in 2018, apalutamide has become a possible treatment option for nmCRPC patients (Smith et al., 2018). Furthermore, two other antitumor agents, namely, darolutamide and enzalutamide, have been reported to enhance metastasis-free survival in nmCRPC patients (Hussain et al., 2018; Fizazi et al., 2019). When compared with chemotherapy, these agents have shown more promising results due to fewer toxicities. However, these studies may have not accurately detected the overall tumor burden due to the low specificity and sensitivity of the CIMs used in these studies. Therefore, more prospective studies are needed to evaluate the treatment options in the patients with “true” nmCRPC with no visible metastases using both the PSMA-based PET/CT and CIMs. Those patients, initially assessed as nmCRPC by using CIMs but restaged as mCRPC by using PSMA-based PET, in theory, are at high risk of early disease progression. Systemic therapies like docetaxel-based chemotherapy should not be delayed in such patients.

### Prostate-specific membrane antigen-based PET/CT may also be useful to serve as a triage tool for castration-resistant prostate cancer patients

Certain treatment options can be excluded based on the PSMA-based PET/CT. For instance, the ^223^Ra-dichloride therapy is eligible only for patients with two or more bone metastases but without known visceral metastases based on cross-sectional imaging techniques, as reported in the ALSYMPCA trial (Parker et al., 2013). The use of PSMA-based PET as a staging tool for CRPC may detect more visceral metastases when compared with the CIMs. Therefore, these newly detected metastases may prohibit a considerable number of patients from receiving the ^223^Ra-dichloride treatment (Bräuer et al., 2017a). Furthermore, for the patients who are potential candidates for PSMA-RLT, PSMA-based PET scans should be used to exclude patients with low or no expression of PSMA (Ost, 2020; Calais and Czernin, 2021; Srinivas and Iagaru, 2021).

PSMA-based PET/CT might serve as a complementary or even independent biomarker of CRPC tumor load and might create new diagnostic criteria for CRPC. Further studies are required to elucidate the accuracy and clinical benefits of PSMA-based imaging in CRPC patients.

### Therapeutic role of prostate-specific membrane antigen-based imaging and prostate-specific membrane antigen-radioligand therapy

#### Prostate-specific membrane antigen-guided metastasis-directed therapy in the oligometastatic castration-resistant prostate cancer patients

The term “oligometastatic disease” was proposed by Hellman and Weichselbaum in 1995, which represents an intermediate state between regionally confined and disseminated malignancies limited in the number (≤5) of metastatic (M1) sites (Rao et al., 2019). In theory, before the occurrence of widespread metastases, the tumor cells may have only restricted metastatic potential. In theory, an aggressive local therapy, such as radiation, before the occurrence of extensive metastases can delay the time for systemic therapies, thereby reducing their relevant toxicities and/or prolonging the progression-free survival (PFS) (Weichselbaum and Hellman, 2011). The accurate visualization of regional or disseminated metastases using PSMA-based PET/CT makes them potential candidates for metastasis-directed therapy (MDT), such as stereotactic body radiotherapy. MDT guided by PSMA-based imaging has shown promising results in patients with hormone-naïve oligometastatic recurrent PCa (Beauval et al., 2018; Kneebone et al., 2018; Ost et al., 2018; Siva et al., 2018). Therefore, the potential role of MDT guided by PSMA-based imaging for patients with oligometastatic CRPC has been focused on.

In 2016, Muldermanns et al. retrospectively reviewed a total of 66 oligometastatic PCa patients, including 50 CRPC patients, who underwent stereotactic body radiation therapy guided by PSMA-PET/CT. They observed a biochemical PFS among 54% of the patients (Muldermans et al., 2016). Guler et al. investigated the efficacy of ^68^Ga-PSMA-11-based PET/CT-guided radiotherapy for the treatment of 23 oligometastatic PCa patients, including nine mCRPC patients. A total of 83% of patients showed remission after a 7-month median follow-up (Guler et al., 2018). Likewise, Lohaus et al. reported longer responses to PSMA-guided local ablative radiotherapy among 15 patients with oligometastatic CRPC. The PSMA-guided local ablative radiotherapy for metastases could achieve a PSA response in most of the patients, thereby delaying the beginning of systemic therapy (Lohaus et al., 2019). In a retrospective clinical trial of 50 patients with oligometastatic PCa, including 15 CRPC, Kalinauskaite and others reported that PSMA-based PET-guided radiotherapy could achieve a high 2-year local control rate (96%) while showing fewer side effects (Kalinauskaite et al., 2020). These results are consistent with the hypothesis that the PSMA-PET/CT can identify the relevant metastases that cannot be effectively controlled by the ADT in the CRPC, indicating that the PSMA-guided MDT, such as radiation and ablation, might be used in oligometastatic CRPC (Muldermans et al., 2016; Guler et al., 2018; Lohaus et al., 2019; Kalinauskaite et al., 2020).

In the past, the intensification of systemic therapy was the standard treatment when PCa progressed into a castration-resistant state. Local radiotherapy was applied with a palliative intention to alleviate or prevent symptoms. The utilization of PSMA-based PET/CT as a staging tool for patients with CRPC may provide higher sensitivity and specificity and precisely localize the lesions when compared with conventional imaging techniques. It can be inferred that the improved accuracy of PSMA-based imaging resulted in a more precise and earlier localization of the oligometastatic lesions, thereby offering an opportunity for the MDT of the oligometastatic CRPC.

At present, the specific effects of PSMA on the therapeutic decision and radiotherapy planning remain unclear in the oligometastatic CRPC. In the reports mentioned above (Muldermans et al., 2016; Guler et al., 2018; Lohaus et al., 2019; Kalinauskaite et al., 2020), the patient backgrounds and radiotherapy regimens were heterogeneous, and follow-up periods were relatively short. Furthermore, the PSA response as the primary endpoint in the above-mentioned reports is not convincing enough because it is not always connected to the clinical PFS or overall survival (OS). Therefore, further prospective interventional trials are needed to compare the clinical benefits difference between PSMA-guided MDTs and systemic treatment for improving the OS and quality of life in oligometastatic CRPC patients.

### Prostate-specific membrane antigen-radioligand therapy

PSMA-RLT has been demonstrated to be an effective and well-tolerated treatment for mCRPC. RLT involves the use of radiolabeled compounds or radioisotopes, which are either natural or designed to accumulate in the targeted cancer cells, delivering a certain amount of radiation to kill the tumor cells and minimize the damage to nearby normal tissues (Czerwi ´nska et al., 2020; Rahbar et al., 2018). The combined treatment of widespread bone and extra-skeletal metastases is now possible due to the systemic administration of tumor-targeted radiopharmaceuticals. This approach spares the normal tissues, avoiding negative impacts (Quast, 2006).

Novel radionuclides have been constantly developed and introduced as RLT in clinical practices for mCRPC treatment. The low‐molecule inhibitors of PSMA have been mostly utilized as radioligands in PSMA-RLT studies due to their lower hematotoxicity when compared with that of the monoclonal antibodies (de Bono et al., 2021). The low‐molecule PSMA ligands have the potential to be labeled with a wide variety of radioisotopes, including alpha and beta emitters. ^177^Lu, a beta emitter, and ^225^Ac, an alpha emitter, are the most commonly used radionuclides for PSMA-RLT (Sgouros et al., 2020). In general, the beta emitters have a lower LET (linear energy transfer, 0.2 keV/m) and a longer emission range (1.5–12 mm), making them more effective in treating medium-to-large tumors. By contrast, the shorter emission range (40–100 µm) and higher LET (80 keV/m) of alpha emitters make them a good candidate for the treatment of microscopic tumor cells (Kassis, 2008; Nelson et al., 2020).

### Efficiency of beta emitters radioligand therapy

^177^Lu has promising physical properties, such as a long half-life of 6.7 days, a short-range medium-energy beta particle for the crossfire to neighboring tumors, and low energy gamma emission for the posttreatment dosimetry analysis (Enger et al., 2008). PSMA-617 is the most frequently used ligand in RLT and can combine with ^177^Lu, forming ^177^Lu-PSMA-617, which is the most clinically used agent in PSMA-RLT (Benešová et al., 2015).

In the last decades, several studies with small patient cohorts showed promising antitumor effects with acceptable toxicity among patients with mCRPC treated with ^177^Lu-PSMA-617 (Ahmadzadehfar et al., 2015; Ahmadzadehfar et al., 2016; Kratochwil et al., 2016a; Baum et al., 2016; Rahbar et al., 2016; Bräuer et al., 2017b; Fendler et al., 2017; Scarpa et al., 2017; Yadav et al., 2017). In a larger multicenter study cohort including 144 mCRPC patients, treated with ^177^Lu-PSMA-617, the investigators reported a decline in the PSA level among 60% of patients and a decline in the PSA levels ≥50% among 45% of patients with acceptable toxicity (Rahbar et al., 2017).

In the first prospective single-arm Phase 2 study (LuPSMA), ^177^Lu-PSMA-617 achieved a decline in the PSA levels ≥50% among 57% of the patients and pain relief, as well as the median PFS and OS of 7.6 and 13.5 months, among the mCRPC patients who had already progressed after conventional therapies (Hofman et al., 2018; Violet et al., 2020). The randomized multicenter Phase II trial TheraP assessed the value of ^177^Lu-PSMA-617 among 200 mCRPC patients, for whom cabazitaxel was considered the next appropriate treatment (de Wit et al., 2019; Hofman et al., 2021). When compared with the cabazitaxel group, the ^177^Lu-PSMA-617 treatment showed a greater PSA response rate (66 vs. 37%). The ^177^Lu-PSMA-617 also showed a more significant delay in the radiographic as well as PSA progression. However, the median PFS between the two groups was similar (Hofman et al., 2021). In addition, a Phase Ⅲ VISION trial reported that the standard of care plus ^177^Lu-PSMA-617 was more effective when compared with the standard of care alone in the patients with mCRPC who progressed after taxane and novel androgen axis therapies. The ^177^Lu-PSMA-617 arm displayed better PFS (8.7 months vs. 3.4 months) and OS (15.3 months vs. 11.3 months) after a median follow-up of 20.9 months (Sartor et al., 2021).

In addition, except for PSMA-617, ^177^Lu was combined with the PSMA imaging and therapeutic ligand (PSMA I&T), showing similar PSMA affinity, dosimetry, and pharmacokinetics to those of ^177^Lu-PSMA-617 (Weineisen et al., 2015). In the largest cohort of mCRPC patients who received ^177^Lu-PSMA I&T therapy (Heck et al., 2019), a decline in the PSA levels by over 50% was observed among 38% of patients, with a PFS and OS of 4.1 and 12.9 months, respectively. SPLASH (NCT04647526) study compared the effectiveness of ^177^Lu-PSMA-I&T to that of enzalutamide or abiraterone in mCRPC patients and the primary results are expected to be reported in 2023. There is no head‐to‐head study of ^177^Lu‐PSMA‐I&T and ^177^Lu‐PSMA‐617 at the moment. However, ^177^Lu-PSMA-617 is preferred over ^177^Lu-PSMA I&T in clinical practices, which might be due to its lower uptake by the kidney (Schuchardt et al., 2021).

Although the difference was not statistically significant, ^177^Lu‐PSMA-RLT had a longer OS when compared with that of the conventional third‐line therapies for the mCRPC patients in a recent systematic review (mean of 14 vs. 12 months, respectively, *p* = 0.32) (von Eyben et al., 2018). Several prospective studies on the mCRPC and metastasis hormone-sensitive prostate cancer (mHSPC) patients in earlier disease settings are in progress, including the neoadjuvant treatment in high risk primary cancers and an earlier line of treatment for mCRPC and mHSPC (NCT04443062, NCT04343885, NCT04720157, NCT04297410, NCT04647526, and NCT04689828), and their results are eagerly awaited.

### Efficiency of alpha emitter radioligand therapy

^177^Lu is the most clinically used radiation source for RLT. However, not all the patients responded to ^177^Lu-labeled PSMA ligands, and the presence of diffuse bone marrow infiltration was still considered a health risk for the hematologically toxic effects despite the overall low toxicity of ^177^Lu (Delker et al., 2016). Because of their higher LET and shorter emission, the alpha emitters specifically target and kill the individual cancer cells more effectively while reducing the toxic hematological effects in patients with diffuse bone marrow infiltration (Kratochwil et al., 2014; Seidl, 2014).

Among the alpha‐emitting radioisotopes, ^225^Ac-PSMA-I&T and ^225^Ac-PSMA-617 are the most investigated radioisotopes and have been proven to be effective in treating mCRPC. Kratochwil et al. published the first case report of ^225^Ac-PSMA-617 therapy in two patients with diffuse bone marrow infiltration and resistance to ^177^Lu-PSMA-617. The PET/CT scan revealed that both the patients had a complete response, and their PSA levels dropped below the detectable levels (Kratochwil et al., 2016b). Subsequent trials confirmed the treatment efficacy and survival benefits of the alpha emitter RLT (Kratochwil et al., 2017; Kratochwil et al., 2018a; Sathekge et al., 2019).

Due to the difference in the micro-dosimetry throughout the cancer cells, the targeted alpha radiotherapy has the potential to eliminate the resistance to beta radiotherapy (Seidl, 2014). The patients, who had previously failed to respond to ^177^Lu-PSMA-RLT treatment could also get an advantage from ^225^Ac-PSMA-617 therapies. Yadav et al. conducted a prospective study, in which ^225^Ac-PSMA-617 was administered to treat mCRPC in a total of 28 male patients (15 of the patients had received prior ^177^Lu-PSMA-RLT); 78.6% of the patients showed a 50% decrease in the PSA levels by the end of the follow-up period (Yadav et al., 2020). Fuerecker et al. investigated the potential of ^225^Ac-PSMA-617 among the mCRPC patients, who progressed after a median of four treatment cycles of ^177^Lu-PSMA. A decrease in the PSA level was observed in 23 of the 26 patients, showing a PSA decline of over 50% in 17 patients (Feuerecker et al., 2021). Khreish and others hypothesized that among the patients, who showed no response to ^177^Lu-PSMA ligand alone, a tandem treatment, consisting of low-activity ^225^Ac-PSMA ligand and high-activity beta emitter ligand, could increase the effectiveness while decreasing the xerostomia severity. Further studies are needed to validate this hypothesis (Khreish et al., 2020).

Although these retrospective studies showed encouraging results, it is necessary to collect additional prospective data about alpha emitters radioligand RLT. Additional studies are needed to explore the intracellular localization, retention times, and stability of ^225^Ac-PSMA complexes destined for the radioligand therapy in cancer and normal tissues, especially in the renal tissues (Kratochwil et al., 2018b), because the excretion of ^225^Ac-PSMA-617 through the kidney might lead to nephrotoxicity (Ristau et al., 2014; Gallyamov et al., 2019). Furthermore, the lack of ^225^Ac availability is a primary obstacle that must be overcome for its use in clinical studies (Robertson et al., 2018).

### Safety of prostate-specific membrane antigen-radioligand therapy

In terms of toxicity, PSMA-RLT has also shown encouraging results. A recent meta-analysis evaluated 36 reported studies and 2,346 patients who received either ^177^Lu or ^225^Ac-RLT and showed that no patients died of severe adverse effects (von Eyben et al., 2020). Less than 1% of the patients had hematologic adverse effects Grade 4 and severe nonhematologic adverse effects. Grade 3 toxicities including anemia, leukopenia, and thrombocytopenia were reported in a median of 10%, 3%, and 2% of the treated patients. In the prospective study of 43 patients with mCRPC treated with ^177^Lu‐PSMA‐617, Grade 3–4 thrombocytopenia was observed in approximately 27% of patients, among which, 13% could possibly be attributed to PSMA-RLT (Hofman et al., 2018; Violet et al., 2020). In the TheraP study Grade 3–4 adverse events were reported in 33% of the patients in the ^177^Lu-PSMA-617 group vs. 53% of the patients in the cabazitaxel group, implying that this novel therapeutic option was less toxic than chemotherapy (Hofman et al., 2021).

The studies of PSMA-RLT reported mild nausea, loss of appetite, and fatigue as the most common but usually transient nonhematologic adverse effects. Despite the physiological expression of PSMA in kidneys, the Grade 3 or 4 renal toxicity in ^177^Lu-RLT has not been reported yet. Xerostomia was also a common side effect in PSMA-RLT especially in alpha emitter RLT (75–78), stopping the ^225^Ac-RLT in 10–25% of the patients. Sialendoscopy with dilatation, steroid injection, saline irrigation, salivary gland cooling externally using ice packs, and intraparenchymal botulinum toxin injections were performed with limited success. Therefore, the reduction of salivary toxicity needs further investigations (Baum et al., 2018; Langbein et al., 2018; Rathke et al., 2019).

### Limitation of prostate-specific membrane antigen-radioligand therapy

A potential response to the PSMA-RLT is predicated based on the elevated PSMA expression of tumor lesions. The interpatient and intrapatient heterogeneities of PSMA expression have been cited as a potential pitfall of PSMA-RLT and might limit its clinical application. PSMA expression might be downregulated by preferential therapy stress, which might result in a limited response to PSMA-RLT in some patients. The significant interpatient and intrapatient heterogeneities of PSMA expression have been noted in immunohistochemistry analysis of mCRPC lesions (Mannweiler et al., 2009). Preclinical studies also suggested that despite an overall increase in the PSMA expression during the progression of PCa from androgen sensitivity to androgen independence, some metastatic cell lines could lose PSMA expression (Laidler et al., 2005; Paschalis et al., 2019). Although most liver metastases express PSMA highly, a significant proportion of liver metastases may also lack PSMA expression (Damjanovic et al., 2019). The heterogeneity of PSMA expression may partly explain why some patients do not respond to PSMA-RLT.

### Prostate-specific membrane antigen-targeted immunotherapy

PSMA has also been the hotspot of targeted immunotherapeutic approaches to mCRPC, such as bispecific T‐cell engager (BiTE). BiTE therapies generally involve a bispecific antibody with two binding sites which target the tumor-associated antigen on tumor cells, besides cluster of differentiation 3 (CD3) on T cells. In doing so, it induces T-cell activation, cytokine production, and T-cell–mediated lysis leading to targeted tumor cell death. PSMA is the perfect candidate antigen for BiTE therapies due to its advantages as described above (O’Keefe et al., 2018; Sweat et al., 1998; Silver et al., 1997; Paschalis et al., 2019; Nguyen et al., 2016). The first generation PSMA/CD3‐bispecific BiTE antibody AMG212 [MT112 (Micromet, Inc.)/BAY2010112 (Bayer AG)] has been found to potently suppress tumor growth in mouth models as well as in a Phase I study involving 47 mCRPC patients (Hummel et al., 2021). To overcome the short half-life of AMG212, the next-generation PSMA-targeted BiTE molecule, AMG 160, was developed and showed promising antitumor activity in mCRPC xenograft models (Deegen et al., 2021). Now, the clinical benefit of AMG 160 is being evaluated in an ongoing Phase I study in mCRPC patients (NCT03792841).

### Prognostic role of prostate-specific membrane antigen-based imaging in patients receiving prostate-specific membrane antigen-radioligand therapy

#### Prostate-specific membrane antigen PET/CT

PSMA-RLT is an emerging treatment option for patients with mCRPC. The identification of patients at risk of poor prognosis is clinically important. Although widely used, the relative changes in conventional imaging and laboratory parameters (mainly serum PSA levels) might not be associated with treatment response or patient outcome. The PSMA-based PET/CT has been widely used to select patients for PSMA-RLT (65,95), and parameters derived from PET/CT scans have been demonstrated to be related to patients’ outcomes receiving PSMA-RLT (31–33).

Standardized uptake value (SUV) is a widely used quantifiable parameter in PET scans and partially reflects the tracer expression *in vivo*. SUV derived from PSMA-PET/CT could represent tumor PSMA expression and therefore be used to predict responses and clinical outcomes. Ferdinandus et al. examined the patients, who were recruited in the LuPSMA trial, to investigate if their baseline imaging had any predictive value. The average intensity of PSMA-avid tumor uptake was significantly correlated with the OS, using a threshold value of SUV of >3 (Ferdinandus et al., 2020). They previously reported that the tracer uptake in PSMA-PET/CT correlated with the PSA response after 12 weeks in the patients treated with PSMA-RLT (Violet et al., 2019). Vlachostergios and others used a five-point imaging score (IS), ranging from 0 to 4, which was assigned based on the comparison of PSMA uptake between tumors and the liver (Vlachostergios et al., 2021). Two experienced radiologists independently scored by comparing the liver SUVmean with averaged SUVmax of the five lesions with the highest uptake. They analyzed 215 mCRPC patients treated with beta-emitting radionuclides and confirmed that a high IS (defined as IS ≥ 2) was independently related to a ≥50% decrease in PSA, after accounting for the CALGB (Halabi) prognostic score (Halabi et al., 2014), dose administration, and the previous use of taxane (Vlachostergios et al., 2021).

Apart from routinely used parameters like SUV, radiomic methods are regarded as efficient, and reliable methods to extract a variety of quantitative features from imaging data. It has been proven to be useful to identify the curative effect and predict outcomes in cancers, such as breast carcinoma (Li et al., 2016). At present, radiomic methods are more frequently applied to CT and MRI than to PET imaging data. The existing reports on radiomic analysis of PSMA-PET imaging data focus mostly on PCa at an earlier stage (Zamboglou et al., 2019). Roll et al. reported the first radiomics analysis of pretherapeutic PSMA-PET scans prior to PSMA-RLT, showing that it may potentially offer predictive and prognostic parameters (Roll et al., 2021). The data on radiomic analysis of PSMA-PET in CRPC patients are still sparse.

A potential response to the PSMA-RLT is predicated based on the elevated PSMA expression. In general, the PSMA expression in PCa lesions increases with tumor dedifferentiation and castration resistance. However, dedifferentiated neuroendocrine PCa may be an exception in which PSMA expression can be low due to the suppression of the PSMA gene (Bakht et al., 2018). Therefore, due to significant interpatient and intrapatient heterogeneity of PSMA expression as described above (Laidler et al., 2005; Mannweiler et al., 2009; Damjanovic et al., 2019), pretreatment PSMA-PET/CT imaging is necessary prior to PSMA-RLT to verify the tumor PSMA expression.

### Dual tracer FDG and prostate-specific membrane antigen- PET/CT

It has been demonstrated that the glucose metabolism of PCa correlated with the cell proliferative activity expressed by the Ki-67 index. This supported the assumption that PCa with higher glucose metabolism might progress rapidly with a poorer prognosis (Agus et al., 1998). Suman et al. reported that high FDG uptake (SUVmax > 15) was associated with a high Gleason score > 8, lack of response, and short PFS (Suman et al., 2019). Numerous clinical studies showed that the prognosis was inversely related to higher glucose metabolism in mCRPC patients (Bauckneht et al., 2021; Wibmer et al., 2021). Recent studies have focused on the combination of FDG and PSMA-PET/CT especially its prognostic value in patients receiving PSMA-RLT.

An assessment of 50 Lu-PSMA trial patients to use both the FDG and PSMA-PET revealed that the patients with limited levels of FDG uptake in disease had a longer OS (Ferdinandus et al., 2020). It was noteworthy that there was no correlation between the patient outcome and the amount of tumor burden as determined via PSMA-PET/CT imaging (Ferdinandus et al., 2020), suggesting that in the stage of advanced mCRPC, the FDG-defined tumor burden might have a greater impact on the patient’s prognosis than PSMA.

Khreish et al. initially described the use of FDG PET/CT in combination with PSMA-PET/CT to distinguish the various phenotypes of lesions (PSMA+/FDG±, FDG+/PSMA−) during ongoing ^177^Lu-PSMA-617-RLT. This retrospective study included 29 patients with mCRPC continuously receiving PSMA-RLT, who underwent both the PSMA and FDG PET/CT scans at the time of the disease progression. In the consolidated PET imaging, 17/29 patients had at least one mismatch metastasis (FDG+/PSMA−) and showed a significantly lower OS of 3.3 months from the time of PET imaging when compared with those without mismatch metastasis. The patients with a higher metabolic tumor volume (MTV, a parameter derived from FDG PET/CT representing tumor glucose metabolism level) had a significantly shorter OS than the patients with a lower MTV while analyzing the subgroup of the patients with mismatch findings (Khreish et al., 2021). Michalski et al. divided 54 mCRPC patients into two groups using dual tracer PET/CT imaging before RLT, including 18 patients with FDG+/PSMA− lesions and 36 patients without FDG+/PSMA− lesions. The patients having one or more FDG+/PSMA− lesions showed a considerably shorter OS (6.0 ± 0.5 months) than the other group (16.0 ± 2.4 months) (Michalski et al., 2021).

Hartrampf et al. tried to evaluate the potential prognostic value of newly found FDG+/PSMA− lesions during the PSMA-RLT. A total of 32 patients, having no FDG+/PSMA− before RLT, underwent the dual tracer PET/CT imaging after two or four cycles of PSMA-RLT. There were no significant differences in the OS among the patients with and without FDG+/PSMA− lesions after two cycles (*p* = 0.807) and four cycles (*p* = 0.442), which might be due to the small sample size. Therefore, the prognosis value of dynamic observation with dual tracer PET/CT is needed to be evaluated in a larger cohort (Hartrampf et al., 2021).

Dual tracer PET/CT of FDG and PSMA allow for the whole-body characterization of the PCa phenotype, which has been beneficial. First, they recognize the tumor sites, which are FDG-positive but PSMA-negative, indicating that they are not susceptible to PSMA-RLT. Second, the combination of FDG and PSMA tracers gives a whole-body scan rather than being limited to the osseous metastasis only. Third, they offer a unique estimation of tumor glycolysis, which is enhanced in the aggressive subtypes of the tumor. It was speculated that in the mCRPC, the tumor cells lose their PSMA expression and glucose reprogrammed with the disease progression, thereby making it more aggressive. Tumors with reduced PSMA uptake and high FDG uptake are aggressive disease sites, which cannot be successfully targeted by PSMA-RLT. From a theoretical perspective, the patients with PSMA-avidity and high FDG-avidity lesions might be more eligible for taxane-based chemotherapies rather than the PSMA-RLT. However, the patients, lacking such lesions, are likely to show a better response to PSMA-RLT and second-generation antiandrogens, which could be considered early in the course of the disease (Basu et al., 2020). How the dual tracer PET/CT can provide insights into the different PCa phenotypes and individualize the decision making in patients need further investigation.

## Conclusion

PSMA-based PET/CT imagings have a higher sensitivity and specificity than conventional imaging techniques, which have the potential to redefine the nmCRPC and mCRPC. PSMA-based PET/CT may serve as a complementary or even independent biomarker of CRPC tumor load. Further studies are needed to elucidate the accuracy and clinical benefits of PSMA-based imagings in CRPC patients. Owing to the accurate location of CRPC lesions, the PSMA-PET can serve as a guide for MDT in patients with oligometastatic CRPC. The PSMA ligands labeled with various alpha or beta emitters radionuclides have a comparative or even better survival benefit than conventional third‐line therapies for mCRPC. However, there is a need to explore strategies to attain deeper and more durable responses. Nonradioactive approaches like BiTE therapies are also of emerging interest. In combination with other radiotracers, such as ^18^F-FDG the PSMA-based imaging can serve as a prognostic tool for mCRPC patients before receiving PSMA-RLT. Radiomic analyses of PSMA-PET scans in CRPC patients are also worthy of more exploration.

## References

[B1] Agus D. B. Golde D. W. Sgouros G. Ballangrud A. Cordon-Cardo C. Scher H. I. (1998). Positron emission tomography of a human prostate cancer xenograft: association of changes in deoxyglucose accumulation with other measures of outcome following androgen withdrawal. Cancer Res. 58 (14), 3009–3014. 9679964

[B2] Ahmadzadehfar H. Eppard E. Kürpig S. Fimmers R. Yordanova A. Schlenkhoff C. D. (2016). Therapeutic response and side effects of repeated radioligand therapy with 177Lu-PSMA-DKFZ-617 of castrate-resistant metastatic prostate cancer. Oncotarget 7 (11), 12477–12488. 10.18632/oncotarget.7245 26871285 PMC4914299

[B3] Ahmadzadehfar H. Rahbar K. Kürpig S. Bögemann M. Claesener M. Eppard E. (2015). Early side effects and first results of radioligand therapy with (177)Lu-DKFZ-617 PSMA of castrate-resistant metastatic prostate cancer: a two-centre study. EJNMMI Res. 5 (1), 114. 10.1186/s13550-015-0114-2 26099227 PMC4477007

[B4] Bakht M. K. Derecichei I. Li Y. Ferraiuolo R. M. Dunning M. Oh S. W. (2018). Neuroendocrine differentiation of prostate cancer leads to PSMA suppression. Endocr. Relat. Cancer 26 (2), 131–146. 10.1530/ERC-18-0226 30400059

[B5] Basu S. Parghane R. V. Suman S. Joshi A. Prabhash K. Bakshi G. (2020). Towards personalizing treatment strategies in mCRPC: Can dual-tracer PET-CT provide insights into tumor biology, guide the optimal treatment sequence, and individualize decision-making (between chemotherapy, second-generation anti-androgens and PSMA-directed radioligand therapy) early in the disease course?. Eur. J. Nucl. Med. Mol. Imaging 47 (8), 1793–1797. 10.1007/s00259-019-04616-w 31776630

[B6] Bauckneht M. Bertagna F. Donegani M. I. Durmo R. Miceli A. De Biasi V. (2021). The prognostic power of 18F-FDG PET/CT extends to estimating systemic treatment response duration in metastatic castration-resistant prostate cancer (mCRPC) patients. Prostate Cancer Prostatic Dis. 24 (4), 1198–1207. 10.1038/s41391-021-00391-8 34012060 PMC8616756

[B7] Baum R. P. Kulkarni H. R. Schuchardt C. Singh A. Wirtz M. Wiessalla S. (2016). 177Lu-Labeled prostate-specific membrane antigen radioligand therapy of metastatic castration-resistant prostate cancer: safety and efficacy. J. Nucl. Med. 57 (7), 1006–1013. 10.2967/jnumed.115.168443 26795286

[B8] Baum R. P. Langbein T. Singh A. Shahinfar M. Schuchardt C. Volk G. F. (2018). Injection of botulinum toxin for preventing salivary gland toxicity after PSMA radioligand therapy: an empirical proof of a promising concept. Nucl. Med. Mol. Imaging 52 (1), 80–81. 10.1007/s13139-017-0508-3 29391917 PMC5777965

[B9] Beauval J. B. Loriot Y. Hennequin C. Rozet F. Barthelemy P. Borchiellini D. (2018). Loco-regional treatment for castration-resistant prostate cancer: is there any rationale? a critical review from the AFU-GETUG. Crit. Rev. Oncol. Hematol. 22, 144–149. 10.1016/j.critrevonc.2017.12.012 29458782

[B10] Benešová M. Schäfer M. Bauder-Wüst U. Afshar-Oromieh A. Kratochwil C. Mier W. (2015). Preclinical evaluation of a tailor-made DOTA-conjugated PSMA inhibitor with optimized linker moiety for imaging and endoradiotherapy of prostate cancer. J. Nucl. Med. 56, 914–920. 10.2967/jnumed.114.147413 25883127

[B11] Bräuer A. Grubert L. S. Roll W. Schrader A. J. Schäfers M. Bögemann M. (2017). ^177^Lu-PSMA-617 radioligand therapy and outcome in patients with metastasized castration-resistant prostate cancer. Eur. J. Nucl. Med. Mol. Imaging 44 (10), 1663–1670. 10.1007/s00259-017-3751-z 28624848

[B12] Bräuer A. Rahbar K. Konnert J. Bögemann M. Stegger L. (2017). Diagnostic value of additional ^68^Ga-PSMA-PET before ^223^Ra-dichloride therapy in patients with metastatic prostate carcinoma. Nuklearmedizin. 56, 14–22. 10.3413/Nukmed-0846-16-09 28074210

[B13] Calais J. Czernin J. (2021). PSMA expression assessed by PET imaging is a required biomarker for selecting patients for any PSMA-targeted therapy. J. Nucl. Med. 62, 1489–1491. 10.2967/jnumed.121.263159 34725231 PMC8612346

[B14] Carlucci G. Ippisch R. Slavik R. Mishoe A. Blecha J. Zhu S. (2021). ^68^Ga-PSMA-11 nda approval: a novel and successful academic partnership. J. Nucl. Med. 62 (2), 149–155. 10.2967/jnumed.120.260455 33443068 PMC8679592

[B15] Cornford P. van den Bergh R. C. N. Briers E. Van den Broeck T. Cumberbatch M. G. De Santis M. (2021). EAU-EANM-ESTRO-ESUR-SIOG guidelines on prostate cancer. Part II-2020 update: treatment of relapsing and metastatic prostate cancer. Eur. Urol. 79 (2), 263–282. 10.1016/j.eururo.2020.09.046 33039206

[B16] Czerwi ´nska M. Bilewicz A. Kruszewski M. Wegierek-Ciuk A. Lankoff A. (2020). Targeted radionuclide therapy of prostate cancer-from basic research to clinical perspectives. Molecules 25, 1743. 10.3390/molecules25071743 PMC7181060 32290196

[B17] Damjanovic J. Janssen J. C. Prasad V. Diederichs G. Walter T. Brenner W. (2019). ^68^Ga-PSMA-PET/CT for the evaluation of liver metastases in patients with prostate cancer. Cancer Imaging 19 (1), 37. 10.1186/s40644-019-0220-x 31186052 PMC6560719

[B18] de Bono J. S. Fleming M. T. Wang J. S. Cathomas R. Miralles M. S. Bothos J. (2021). Phase I study of MEDI3726: a prostate-specific membrane antigen-targeted antibody-drug conjugate, in patients with mCRPC after failure of abiraterone or enzalutamide. Clin. Cancer Res. 27 (13), 3602–3609. 10.1158/1078-0432.CCR-20-4528 33795255

[B19] de Wit R. de Bono J. Sternberg C. N. Fizazi K. Tombal B. Wülfing C. (2019). Cabazitaxel versus abiraterone or enzalutamide in metastatic prostate cancer. N. Engl. J. Med. 381, 2506–2518. 10.1056/NEJMoa1911206 31566937

[B20] Deegen P. Thomas O. Nolan-Stevaux O. Li S. Wahl J. Bogner P. (2021). The PSMA-targeting half-life extended BiTE therapy AMG 160 has potent antitumor activity in preclinical models of metastatic castration-resistant prostate cancer. Clin. Cancer Res. 27 (10), 2928–2937. 10.1158/1078-0432.CCR-20-3725 33504551

[B21] Delker A. Fendler W. P. Kratochwil C. Brunegraf A. Gosewisch A. Gildehaus F. J. (2016). Dosimetry for (177)Lu-DKFZ-PSMA-617: a new radiopharmaceutical for the treatment of metastatic prostate cancer. Eur. J. Nucl. Med. Mol. Imaging 43, 42–51. 10.1007/s00259-015-3174-7 26318602

[B22] Enger S. A. Hartman T. Carlsson J. Lundqvist H. (2008). Cross-fire doses from beta-emitting radionuclides in targeted radiotherapy. a theoretical study based on experimentally measured tumor characteristics. Phys. Med. Biol. 53, 1909–1920. 10.1088/0031-9155/53/7/007 18364546

[B23] Fanti S. Hadaschik B. Herrmann K. (2020). Proposal for systemic-therapy response-assessment criteria at the time of PSMA PET/CT imaging: the PSMA PET progression criteria. J. Nucl. Med. 61 (5), 678–682. 10.2967/jnumed.119.233817 31806774 PMC7198387

[B24] Fendler W. P. Calais J. Eiber M. Flavell R. R. Mishoe A. Feng F. Y. (2019). Assessment of 68Ga-PSMA-11 PET accuracy in localizing recurrent prostate cancer: a prospective single-arm clinical trial. JAMA Oncol. 5 (6), 856–863. 10.1001/jamaoncol.2019.0096 30920593 PMC6567829

[B25] Fendler W. P. Reinhardt S. Ilhan H. Delker A. Böning G. Gildehaus F. J. (2017). Preliminary experience with dosimetry, response and patient reported outcome after 177Lu-PSMA-617 therapy for metastatic castration-resistant prostate cancer. Oncotarget 8 (2), 3581–3590. 10.18632/oncotarget.12240 27683041 PMC5356905

[B26] Fendler W. P. Weber M. Iravani A. Hofman M. S. Calais J. Czernin J. (2019). Prostate-specific membrane antigen ligand positron emission tomography in men with non-metastatic castration-resistant prostate cancer. Clin. Cancer Res. 25, 7448–7454. 10.1158/1078-0432.CCR-19-1050 31511295

[B27] Ferdinandus J. Violet J. Sandhu S. Hicks R. J. Ravi Kumar A. S. Iravani A. (2020). Prognostic biomarkers in men with metastatic castration-resistant prostate cancer receiving [177Lu]-PSMA-617. Eur. J. Nucl. Med. Mol. Imaging 47 (10), 2322–2327. 10.1007/s00259-020-04723-z 32140802

[B28] Feuerecker B. Tauber R. Knorr K. Heck M. Beheshti A. Seidl C. (2021). Activity and adverse events of actinium-225-PSMA-617 in advanced metastatic castration-resistant prostate cancer after failure of lutetium-177-PSMA. Eur. Urol. 79 (3), 343–350. 10.1016/j.eururo.2020.11.013 33293081

[B29] Fizazi K. Shore N. Tammela T. L. Ulys A. Vjaters E. Polyakov S. (2019). Darolutamide in nonmetastatic, castration-resistant prostate cancer. N. Engl. J. Med. 380 (13), 1235–1246. 10.1056/NEJMoa1815671 30763142

[B30] Fourquet A. Aveline C. Cussenot O. Créhange G. Montravers F. Talbot J. N. (2020). ^68^Ga-PSMA-11 PET/CT in restaging castration-resistant nonmetastatic prostate cancer: detection rate, impact on patients' disease management and adequacy of impact. Sci. Rep. 10, 2104. 10.1038/s41598-020-58975-8 32034191 PMC7005887

[B31] Gallyamov M. Meyrick D. Barley J. Lenzo N. (2019). Renal outcomes of radioligand therapy: Experience of ^177^lutetium-prostate-specific membrane antigen ligand therapy in metastatic castrate-resistant prostate cancer. Clin. Kidney J. 13 (6), 1049–1055. 10.1093/ckj/sfz101 33391748 PMC7769531

[B32] Guler O. C. Engels B. Onal C. Everaert H. Van den Begin R. Gevaert T. (2018). The feasibility of prostate-specific membrane antigen positron emission tomography(PSMA PET/CT)-guided radiotherapy in oligometastatic prostate cancer patients. Clin. Transl. Oncol. 20, 484–490. 10.1007/s12094-017-1736-9 28795303

[B33] Halabi S. Lin C. Y. Kelly W. K. Fizazi K. S. Moul J. W. Kaplan E. B. (2014). Updated prognostic model for predicting overall survival in first-line chemotherapy for patients with metastatic castration-resistant prostate cancer. J. Clin. Oncol. 32 (7), 671–677. 10.1200/JCO.2013.52.3696 24449231 PMC3927736

[B34] Hansson N. Moll F. Schultheiss D. Krischel M. (2016). Remembering charles B. Huggins' nobel prize for hormonal treatment of prostatic cancer at its 50th anniversary. Eur. Urol. 69 (6), 971–972. 10.1016/j.eururo.2016.01.030 26838478

[B35] Hartrampf P. E. Lapa C. Serfling S. E. Buck A. K. Seitz A. K. Meyer P. T. (2021). Development of discordant hypermetabolic prostate cancer lesions in the course of [^177^Lu]PSMA radioligand therapy and their possible influence on patient outcome. Cancers (Basel) 13 (17), 4270. 10.3390/cancers13174270 34503080 PMC8428347

[B36] He L. Fang H. Chen C. Wu Y. Wang Y. Ge H. (2020). Metastatic castration-resistant prostate cancer: academic insights and perspectives through bibliometric analysis. Med. Baltim. 99 (15), e19760. 10.1097/MD.0000000000019760 PMC7220391 32282738

[B37] Heck M. M. Tauber R. Schwaiger S. Retz M. D'Alessandria C. Maurer T. (2019). Treatment outcome, toxicity, and predictive factors for radioligand therapy with ^177^Lu-PSMA-I&T in metastatic castration-resistant prostate cancer. Eur. Urol. 75, 920–926. 10.1016/j.eururo.2018.11.016 30473431

[B38] Hofman M. S. Emmett L. Sandhu S. Iravani A. Joshua A. M. Goh J. C. (2021). [^177^Lu]Lu-PSMA-617 versus cabazitaxel in patients with metastatic castration-resistant prostate cancer (TheraP): a randomised, open-label, phase 2 trial. Lancet 397, 797–804. 10.1016/S0140-6736(21)00237-3 33581798

[B39] Hofman M. S. Lawrentschuk N. Francis R. J. Tang C. Vela I. Thomas P. (2020). Prostate-specific membrane antigen PET-CT in patients with high-risk prostate cancer before curative-intent surgery or radiotherapy (proPSMA): a prospective, randomised, multicentre study. Lancet 11 (10231), 1208–1216. 10.1016/S0140-6736(20)30314-7 32209449

[B40] Hofman M. S. Violet J. Hicks R. J. Ferdinandus J. Thang S. P. Akhurst T. (2018). [^177^Lu]-PSMA-617 radionuclide treatment in patients with metastatic castration-resistant prostate cancer (LuPSMA trial): a single-centre, single-arm, phase 2 study. Lancet. Oncol. 19, 825–833. 10.1016/S1470-2045(18)30198-0 29752180

[B41] Hovels A. M. Heesakkers R. A. Adang E. M. Jager G. J. Strum S. Hoogeveen Y. L. (2008). The diagnostic accuracy of CT and MRI in the staging of pelvic lymph nodes in patients with prostate cancer:a meta-analysis. Clin. Radiol. 63 (4), 387–395. 10.1016/j.crad.2007.05.022 18325358

[B42] Hummel H. D. Kufer P. Grüllich C. Seggewiss-Bernhardt R. Deschler-Baier B. Chatterjee M. (2021). Pasotuxizumab, a BiTE^®^ immune therapy for castration-resistant prostate cancer: phase I, dose-escalation study findings. Immunotherapy 13 (2), 125–141. 10.2217/imt-2020-0256 33172323

[B43] Hussain M. Fizazi K. Saad F. Rathenborg P. Shore N. Ferreira U. (2018). Enzalutamide in men with nonmetastatic, castration-resistant prostate cancer. N. Engl. J. Med. 378 (26), 2465–2474. 10.1056/NEJMoa1800536 29949494 PMC8288034

[B44] Kalinauskaite G. Senger C. Kluge A. Furth C. Kufeld M. Tinhofer I. (2020). 68Ga-PSMA-PET/CT-based radiosurgery and stereotactic body radiotherapy for oligometastatic prostate cancer. PLoS One 15, e0240892. 10.1371/journal.pone.0240892 33085712 PMC7577453

[B45] Kassis A. I. (2008). Therapeutic radionuclides: biophysical and radiobiologic principles. Semin. Nucl. Med. 38, 358–366. 10.1053/j.semnuclmed.2008.05.002 18662557 PMC2584872

[B46] Khreish F. Ebert N. Ries M. Maus S. Rosar F. Bohnenberger H. (2020). ^225^Ac-PSMA-617/^177^Lu-PSMA-617 tandem therapy of metastatic castration-resistant prostate cancer: Pilot experience. Eur. J. Nucl. Med. Mol. Imaging 47 (3), 721–728. 10.1007/s00259-019-04612-0 31758224

[B47] Khreish F. Ribbat K. Bartholomä M. Maus S. Stemler T. Hierlmeier I. (2021). Value of combined PET imaging with [^18^F]FDG and [^68^Ga]Ga-PSMA-11 in mCRPC patients with worsening disease during [^177^Lu]Lu-PSMA-617 RLT. Cancers (Basel) 13 (16), 4134. 10.3390/cancers13164134 34439287 PMC8391978

[B48] Kneebone A. Hruby G. Ainsworth H. Byrne K. Brown C. Guo L. (2018). Stereotactic body radiotherapy for oligometastatic prostate cancer detected via prostate-specific membrane antigen positron emission tomography. Eur. Urol. Oncol. 1, 531–537. 10.1016/j.euo.2018.04.017 31158100

[B49] Kratochwil C. Bruchertseifer F. Giesel F. L. Weis M. Verburg F. A. Mottaghy F. (2016). 225Ac-PSMA-617 for PSMA-targeted α-radiation therapy of metastatic castration-resistant prostate cancer. J. Nucl. Med. 57 (12), 1941–1944. 10.2967/jnumed.116.178673 27390158

[B50] Kratochwil C. Bruchertseifer F. Rathke H. Bronzel M. Apostolidis C. Weichert W. (2017). Targeted α-therapy of metastatic castration-resistant prostate cancer with ^225^Ac-PSMA-617: dosimetry estimate and empiric dose finding. J. Nucl. Med. 58 (10), 1624–1631. 10.2967/jnumed.117.191395 28408529

[B51] Kratochwil C. Bruchertseifer F. Rathke H. Hohenfellner M. Giesel F. L. Haberkorn U. (2018). Targeted α-therapy of metastatic castration-resistant prostate cancer with ^225^Ac-PSMA-617: swimmer-plot analysis suggests efficacy regarding duration of tumor control. J. Nucl. Med. 59 (5), 795–802. 10.2967/jnumed.117.203539 29326358

[B52] Kratochwil C. Giesel F. L. Bruchertseifer F. Mier W. Apostolidis C. Boll R. (2014). ²¹³Bi-DOTATOC receptor-targeted alpha-radionuclide therapy induces remission in neuroendocrine tumours refractory to beta radiation: a first-in-human experience. Eur. J. Nucl. Med. Mol. Imaging 41 (11), 2106–2119. 10.1007/s00259-014-2857-9 25070685 PMC4525192

[B53] Kratochwil C. Giesel F. L. Stefanova M. Benešová M. Bronzel M. Afshar-Oromieh A. (2016). PSMA-targeted radionuclide therapy of metastatic castration-resistant prostate cancer with 177Lu-labeled PSMA-617. J. Nucl. Med. 57 (8), 1170–1176. 10.2967/jnumed.115.171397 26985056

[B54] Kratochwil C. Schmidt K. Afshar-Oromieh A. Bruchertseifer F. Rathke H. Morgenstern A. (2018). Targeted alpha therapy of mCRPC: Dosimetry estimate of ^213^Bismuth-PSMA-617. Eur. J. Nucl. Med. Mol. Imaging 45 (1), 31–37. 10.1007/s00259-017-3817-y 28891033 PMC5700223

[B55] Laidler P. Dulińska J. Lekka M. Lekki J. (2005). Expression of prostate specific membrane antigen in androgen-independent prostate cancer cell line PC-3. Arch. Biochem. Biophys. 435 (1), 1–14. 10.1016/j.abb.2004.12.003 15680901

[B56] Langbein T. Chaussé G. Baum R. P. (2018). Salivary gland toxicity of PSMA radioligand therapy: relevance and preventive strategies. J. Nucl. Med. 59 (8), 1172–1173. 10.2967/jnumed.118.214379 29903929

[B57] Li H. Zhu Y. Burnside E. S. Drukker K. Hoadley K. A. Fan C. (2016). MR imaging radiomics signatures for predicting the risk of breast cancer recurrence as given by research versions of mamma Print, oncotype DX, and PAM50 gene assays. Radiology 281 (2), 382–391. 10.1148/radiol.2016152110 27144536 PMC5069147

[B58] Lohaus F. Zöphel K. Löck S. Wirth M. Kotzerke J. Krause M. (2019). Can local ablative radiotherapy revert castration-resistant prostate cancer to an earlier stage of disease? Eur. Urol. 75, 548–551. 10.1016/j.eururo.2018.11.050 30578119

[B59] Mannweiler S. Amersdorfer P. Trajanoski S. Terrett J. A. King D. Mehes G. (2009). Heterogeneity of prostate-specific membrane antigen (PSMA) expression in prostate carcinoma with distant metastasis. Pathol. Oncol. Res. 15 (2), 167–172. 10.1007/s12253-008-9104-2 18802790

[B60] Michalski K. Ruf J. Goetz C. Seitz A. K. Buck A. K. Lapa C. (2021). Prognostic implications of dual tracer PET/CT: PSMA ligand and [^18^F]FDG PET/CT in patients undergoing [^177^Lu]PSMA radioligand therapy. Eur. J. Nucl. Med. Mol. Imaging 48 (6), 2024–2030. 10.1007/s00259-020-05160-8 33336265 PMC8113196

[B61] Muldermans J. L. Romak L. B. Kwon E. D. Park S. S. Olivier K. R. (2016). Stereotactic body radiation therapy for oligometastatic prostate cancer. Int. J. Radiat. Oncol. Biol. Phys. 95, 696–702. 10.1016/j.ijrobp.2016.01.032 27131082 PMC5154616

[B62] Nelson B. J. B. Andersson J. D. Wuest F. (2020). Targeted alpha therapy: progress in radionuclide production, radiochemistry, and applications. Pharmaceutics 13 (1), 49. 10.3390/pharmaceutics13010049 33396374 PMC7824049

[B63] Nguyen D. P. Xiong P. L. Liu H. Pan S. Leconet W. Navarro V. (2016). Induction of PSMA and internalization of an anti-PSMA mAb in the vascular compartment. Mol. Cancer Res. 14 (11), 1045–1053. 10.1158/1541-7786.MCR-16-0193 27458033

[B64] O'Keefe D. S. Bacich D. J. Huang S. S. Heston W. D. W. (2018). A perspective on the evolving story of PSMA biology, PSMA-based imaging, and endoradiotherapeutic strategies. J. Nucl. Med. 59 (7), 1007–1013. 10.2967/jnumed.117.203877 29674422 PMC6910646

[B65] Ost P. (2020). PSMA PET-CT redefines nonmetastatic castration-resistant prostate cancer. Nat. Rev. Urol. 17, 133–134. 10.1038/s41585-019-0268-1 31819257

[B66] Ost P. Reynders D. Decaestecker K. Fonteyne V. Lumen N. De Bruycker A. (2018). Surveillance or metastasis-directed therapy for oligometastatic prostate cancer recurrence: a prospective, randomized, multicenter phase II trial. J. Clin. Oncol. 36, 446–453. 10.1200/JCO.2017.75.4853 29240541

[B67] Pandit-Taskar N. O'Donoghue J. A. Durack J. C. Lyashchenko S. K. Cheal S. M. Beylergil V. (2015). A phase I/II study for analytic validation of 89Zr-J591 ImmunoPET as a molecular imaging agent for metastatic prostate cancer. Clin. Cancer Res. 21 (23), 5277–5285. 10.1158/1078-0432.CCR-15-0552 26175541 PMC4668231

[B68] Parker C. Nilsson S. Heinrich D. Helle S. I. O'Sullivan J. M. Fosså S. D. (2013). Alpha emitter radium-223 and survival in metastatic prostate cancer. N. Engl. J. Med. 369, 213–223. 10.1056/NEJMoa1213755 23863050

[B69] Paschalis A. Sheehan B. Riisnaes R. Rodrigues D. N. Gurel B. Bertan C. (2019). Prostate-specific membrane antigen heterogeneity and DNA repair defects in prostate cancer. Eur. Urol. 76 (4), 469–478. 10.1016/j.eururo.2019.06.030 31345636 PMC6853166

[B70] Pyka T. Okamoto S. Dahlbender M. Tauber R. Retz M. Heck M. (2016). Comparison of bone scintigraphy and ^68^Ga-PSMA PET for skeletal staging in prostate cancer. Eur. J. Nucl. Med. Mol. Imaging 43 (12), 2114–2121. 10.1007/s00259-016-3435-0 27290607

[B71] Quast U. (2006). Whole body radiotherapy: A TBI-guideline. J. Med. Phys. 31, 5–12. 10.4103/0971-6203.25664 21206634 PMC3003894

[B72] Rahbar K. Ahmadzadehfar H. Kratochwil C. Haberkorn U. Schäfers M. Essler M. (2017). German multicenter study investigating 177Lu-PSMA-617 radioligand therapy in advanced prostate cancer patients. J. Nucl. Med. 58, 85–90. 10.2967/jnumed.116.183194 27765862

[B73] Rahbar K. Bode A. Weckesser M. Avramovic N. Claesener M. Stegger L. (2016). Radioligand therapy with 177Lu-PSMA-617 as a novel therapeutic option in patients with metastatic castration resistant prostate cancer. Clin. Nucl. Med. 41 (7), 522–528. 10.1097/RLU.0000000000001240 27088387

[B74] Rahbar K. Afshar-Oromieh A. Jadvar H. Ahmadzadehfar H. (2018). PSMA theranostics: current status and future directions. Mol. Imaging 17, 1536012118776068. 10.1177/1536012118776068 29873291 PMC5992796

[B75] Rao A. Vapiwala N. Schaeffer E. M. Ryan C. J. (2019). Oligometastatic prostate cancer: a shrinking subset or an opportunity for cure? Am. Soc. Clin. Oncol. Educ. Book. 39, 309–320. 10.1200/EDBK_239041 31099652

[B76] Rathke H. Kratochwil C. Hohenberger R. Giesel F. L. Bruchertseifer F. Flechsig P. (2019). Initial clinical experience performing sialendoscopy for salivary gland protection in patients undergoing ^225^Ac-PSMA-617 RLT. Eur. J. Nucl. Med. Mol. Imaging 46 (1), 139–147. 10.1007/s00259-018-4135-8 30151743

[B77] Ristau B. T. O'Keefe D. S. Bacich D. J. (2014). The prostate-specific membrane antigen: lessons and current clinical implications from 20 years of research. Urol. Oncol. 32 (3), 272–279. 10.1016/j.urolonc.2013.09.003 24321253 PMC3972351

[B78] Robertson A. K. H. Ramogida C. F. Schaffer P. Radchenko V. (2018). Development of ^225^Ac radiopharmaceuticals: TRIUMF perspectives and experiences. Curr. Radiopharm. 11 (3), 156–172. 10.2174/1874471011666180416161908 29658444 PMC6249690

[B79] Roll W. Schindler P. Masthoff M. Seifert R. Schlack K. Bögemann M. (2021). Evaluation of ^68^Ga-PSMA-11 PET-MRI in patients with advanced prostate cancer receiving ^177^Lu-PSMA-617 therapy: a radiomics analysis. Cancers (Basel) 13 (15), 3849. 10.3390/cancers13153849 34359750 PMC8345703

[B80] Rowe S. P. Macura K. J. Ciarallo A. Mena E. Blackford A. Nadal R. (2016). Comparison of prostate-specific membrane antigen-based 18F-dcfbc PET/CT to conventional imaging modalities for detection of hormone-naïve and castration-resistant metastatic prostate cancer. J. Nucl. Med. 57 (1), 46–53. 10.2967/jnumed.115.163782 26493203 PMC4730886

[B81] Sartor O. de Bono J. Chi K. N. Fizazi K. Herrmann K. Rahbar K. (2021). Lutetium-177-PSMA-617 for metastatic castration-resistant prostate cancer. N. Engl. J. Med. 385, 1091–1103. 10.1056/NEJMoa2107322 34161051 PMC8446332

[B82] Sathekge M. Bruchertseifer F. Knoesen O. Reyneke F. Lawal I. Lengana T. (2019). ^225^Ac-PSMA-617 in chemotherapy-naive patients with advanced prostate cancer: a pilot study. Eur. J. Nucl. Med. Mol. Imaging 46 (1), 129–138. 10.1007/s00259-018-4167-0 30232539 PMC6267694

[B83] Scarpa L. Buxbaum S. Kendler D. Fink K. Bektic J. Gruber L. (2017). The ^68^Ga/^177^Lu theragnostic concept in PSMA targeting of castration-resistant prostate cancer: correlation of SUV_max_ values and absorbed dose estimates. Eur. J. Nucl. Med. Mol. Imaging 44 (5), 788–800. 10.1007/s00259-016-3609-9 28083690

[B84] Scher H. I. Morris M. J. Stadler W. M. Higano C. Basch E. Fizazi K. (2016). Trial design and objectives for castration-resistant prostate cancer: updated recommendations from the prostate cancer clinical trials working group 3. J. Clin. Oncol. 34 (12), 1402–1418. 10.1200/JCO.2015.64.2702 26903579 PMC4872347

[B85] Schuchardt C. Zhang J. Kulkarni H. R. Chen X. Mueller D. Baum R. P. (2021)). Prostate-specific membrane antigen radioligand therapy using ^177^Lu-PSMA I&T and ^177^Lu-PSMA-617 in patients with metastatic castration-resistant prostate cancer: comparison of safety, biodistribution and dosimetry. J. Nucl. Med. 121, 262713. 10.2967/jnumed.121.262713 PMC9364353 34887335

[B86] Schwartz L. H. Seymour L. Litière S. Ford R. Gwyther S. Mandrekar S. (2016). Recist 1.1 - standardisation and disease-specific adaptations: perspectives from the RECIST working group. Eur. J. Cancer 62, 138–145. 10.1016/j.ejca.2016.03.082 27237360 PMC5737786

[B87] Seidl C. (2014). Radioimmunotherapy with α-particle-emitting radionuclides. Immunotherapy 6, 431–458. 10.2217/imt.14.16 24815783

[B88] Sgouros G. Bodei L. McDevitt M. R. Nedrow J. R. (2020). Radiopharmaceutical therapy in cancer: clinical advances and challenges. Nat. Rev. Drug Discov. 19 (9), 589–608. 10.1038/s41573-020-0073-9 32728208 PMC7390460

[B89] Shafi A. A. Yen A. E. Weigel N. L. (2013). Androgen receptors in hormone-dependent and castration-resistant prostate cancer. Pharmacol. Ther. 140 (3), 223–238. 10.1016/j.pharmthera.2013.07.003 23859952

[B90] Silver D. A. Pellicer I. Fair W. R. Heston W. D. Cordon-Cardo C. (1997). Prostate-specific membrane antigen expression in normal and malignant human tissues. Clin. Cancer Res. 3 (1), 81–85. 9815541

[B91] Siva S. Bressel M. Murphy D. G. Shaw M. Chander S. Violet J. (2018). Stereotactic abative body radiotherapy (sabr) for oligometastatic prostate cancer: a prospective clinical trial. Eur. Urol. 74, 455–462. 10.1016/j.eururo.2018.06.004 30227924

[B92] Smith M. R. Saad F. Chowdhury S. Oudard S. Hadaschik B. A. Graff J. N. (2018). Apalutamide treatment and metastasis-free survival in prostate cancer. N. Engl. J. Med. 378 (15), 1408–1418. 10.1056/NEJMoa1715546 29420164

[B93] Song H. Iagaru A. Rowe S. P. (2022). ^18^F-DCFPyL PET acquisition, interpretation, and reporting: suggestions after Food and drug administration approval. J. Nucl. Med. 63 (6), 855–859. 10.2967/jnumed.121.262989 34531266

[B94] Srinivas S. Iagaru A. (2021). To scan or not to scan: an unnecessary dilemma for PSMA radioligand therapy. J. Nucl. Med. 62, 1487–1488. 10.2967/jnumed.121.263035 34446452 PMC8612330

[B95] Suman S. Parghane R. V. Joshi A. Prabhash K. Bakshi G. Talole S. (2019). Therapeutic efficacy, prognostic variables and clinical outcome of ^177^Lu-PSMA-617 PRLT in progressive mCRPC following multiple lines of treatment: prognostic implications of high FDG uptake on dual tracer PET-CT vis-à-vis Gleason score in such cohort. Br. J. Radiol. 92 (1104), 20190380. 10.1259/bjr.20190380 31600089 PMC6913363

[B96] Sung H. Ferlay J. Siegel R. L. Laversanne M. Soerjomataram I. Jemal A. (2021). Global cancer statistics 2020: GLOBOCAN estimates of incidence and mortality worldwide for 36 cancers in 185 countries. Ca. Cancer J. Clin. 71 (3), 209–249. 10.3322/caac.21660 33538338

[B97] Sweat S. D. Pacelli A. Murphy G. P. Bostwick D. G. (1998). Prostate-specific membrane antigen expression is greatest in prostate adenocarcinoma and lymph node metastases. Urology 52 (4), 637–640. 10.1016/s0090-4295(98)00278-7 9763084

[B98] Violet J. Jackson P. Ferdinandus J. Sandhu S. Akhurst T. Iravani A. (2019). Dosimetry of ^177^Lu-PSMA-617 in metastatic castration-resistant prostate cancer: correlations between pretherapeutic imaging and whole-body tumor dosimetry with treatment outcomes. J. Nucl. Med. 60 (4), 517–523. 10.2967/jnumed.118.219352 30291192

[B99] Violet J. Sandhu S. Iravani A. Ferdinandus J. Thang S. P. Kong G. (2020). Long-term follow-up and outcomes of retreatment in an expanded 50-patient single-center phase II prospective trial of ^177^Lu-PSMA-617 theranostics in metastatic castration-resistant prostate cancer. J. Nucl. Med. 61, 857–865. 10.2967/jnumed.119.236414 31732676 PMC7262220

[B100] Vlachostergios P. J. Niaz M. J. Skafida M. Mosallaie S. A. Thomas C. Christos P. J. (2021). Imaging expression of prostate-specific membrane antigen and response to PSMA-targeted β-emitting radionuclide therapies in metastatic castration-resistant prostate cancer. Prostate 81 (5), 279–285. 10.1002/pros.24104 33465252 PMC7904644

[B101] von Eyben F. E. Bauman G. von Eyben R. Rahbar K. Soydal C. Haug A. R. (2020). Optimizing PSMA radioligand therapy for patients with metastatic castration-resistant prostate cancer. a systematic review and meta-analysis. Int. J. Mol. Sci. 21 (23), 9054. 10.3390/ijms21239054 PMC7730994 33260535

[B102] von Eyben F. E. Roviello G. Kiljunen T. Uprimny C. Virgolini I. Kairemo K. (2018). Third-line treatment and ^177^Lu-PSMA radioligand therapy of metastatic castration-resistant prostate cancer: a systematic review. Eur. J. Nucl. Med. Mol. Imaging 45 (3), 496–508. 10.1007/s00259-017-3895-x 29247284 PMC5787223

[B103] Weber M. Kurek C. Barbato F. Eiber M. Maurer T. Nader M. (2021). PSMA-ligand PET for early castration-resistant prostate cancer: a retrospective single-center study. J. Nucl. Med. 62, 88–91. 10.2967/jnumed.120.245456 32444377

[B104] Weichselbaum R. R. Hellman S. (2011). Oligometastases revisited. Nat. Rev. Clin. Oncol. 8, 378–382. 10.1038/nrclinonc.2011.44 21423255

[B105] Weineisen M. Schottelius M. Simecek J. Baum R. P. Yildiz A. Beykan S. (2015). 68Ga- and 177Lu-labeled PSMA I&T: Optimization of a PSMA-targeted theranostic concept and first proof-of-concept human studies. J. Nucl. Med. 56, 1169–1176. 10.2967/jnumed.115.158550 26089548

[B106] Wibmer A. G. Morris M. J. Gonen M. Zheng J. Hricak H. Larson S. (2021). Quantification of metastatic prostate cancer whole-body tumor burden with ^18^F-FDG PET parameters and associations with overall survival after first-line abiraterone or enzalutamide: a single-center retrospective cohort study. J. Nucl. Med. 62 (8), 1050–1056. 10.2967/jnumed.120.256602 33419944 PMC8833874

[B107] Yadav M. P. Ballal S. Sahoo R. K. Tripathi M. Seth A. Bal C. (2020). Efficacy and safety of ^225^Ac-PSMA-617 targeted alpha therapy in metastatic castration-resistant prostate cancer patients. Theranostics 10 (20), 9364–9377. 10.7150/thno.48107 32802197 PMC7415797

[B108] Yadav M. P. Ballal S. Tripathi M. Damle N. A. Sahoo R. K. Seth A. (2017). ^177^Lu-DKFZ-PSMA-617 therapy in metastatic castration resistant prostate cancer: safety, efficacy, and quality of life assessment. Eur. J. Nucl. Med. Mol. Imaging 44 (1), 81–91. 10.1007/s00259-016-3481-7 27506431

[B109] Zamboglou C. Carles M. Fechter T. Kiefer S. Reichel K. Fassbender T. F. (2019). Radiomic features from PSMA PET for non-invasive intraprostatic tumor discrimination and characterization in patients with intermediate- and high-risk prostate cancer - a comparison study with histology reference. Theranostics 9 (9), 2595–2605. 10.7150/thno.32376 31131055 PMC6525993

